# IgG4-Related Disease Retroperitoneal Fibrosis: An Unusual Cause of Low Back Pain

**DOI:** 10.7759/cureus.13608

**Published:** 2021-02-28

**Authors:** Ayrton I Bangolo, Kush Gupta, Adam Atoot

**Affiliations:** 1 Internal Medicine, Hackensack University Medical Center - Palisades Medical Center, North Bergen, USA; 2 Medicine, Kasturba Medical College, Mangalore, IND

**Keywords:** retroperitoneal mass, retroperitoneal fibrosis, back pain, unexplained syncope

## Abstract

IgG4-related disease (IgG4-RD) is a cluster of rare fibroinflammatory diseases that more commonly affect organs such as major salivary glands, biliary tree, periorbital tissues, kidneys, lungs, lymph nodes, retroperitoneum, and less frequently, meninges, aorta, prostate, thyroid gland, pericardium, and the skin. The clinical picture mainly depends on the affected organ and the effects on the surrounding organs, however, the histopathologic findings are very similar regardless of the organ affected. Most patients have a subclinical presentation of the disease and the only clinical manifestation is related to the anatomic location of the disease, whereas some patients may have constitutional symptoms such as weight loss and are often misdiagnosed as having other pathologies (i.e., malignancies, other inflammatory conditions, etc.). Up to 40 percent of patients can have symptoms of allergy or asthma. Patients often have diseases confined to one organ but multiorgan involvement is not uncommon. Patients with multiple organs involvement can have an elevation of up to 30-40 upper limit of normal serum IgG4 concentration; patients with fewer organ involvement can have normal serum IgG4 concentration despite histopathologic findings of the disease.

Idiopathic retroperitoneal fibrosis (RPF) is a commonly encountered subtype of IgG4-RD. Idiopathic retroperitoneal fibrosis accounts for approximately 70 percent of cases and can be divided into IgG4-RD and non-IgG4-RD. Most cases of RPF are incidental findings on radiology studies but should be suspected in any patients complaining of back pain and flank pain, with new-onset kidney dysfunction.

## Introduction

Retroperitoneal fibrosis is one the most common presentation of IgG4-related disease (IgG4-RD). Patients with IgG4-RD retroperitoneal fibrosis have higher chances to have a normal IgG4 serum level, given that the disease is often diagnosed late, in the fibrotic stage. IgG4 serum levels often do not correlate with the disease activity as the level can remain high even in patients with clinical remission; however, patients with rapidly increasing serum IgG4 level can be identified as being at high risk for flare-ups, although flare-ups have also been reported in the patents with normal serum IgG4 levels [1].

In contrast to other autoimmune diseases that mainly affect women of childbearing age, IgG4-RD has a propensity to affect middle-aged to elder men. Pediatric cases have also been reported with similar manifestations as in adults. The prevalence of the disease remains unknown because the disease is often misdiagnosed. Idiopathic retroperitoneal fibrosis (RPF) is a commonly encountered subtype of IgG4-RD [1]. Idiopathic retroperitoneal fibrosis accounts for approximately 70 percent of the cases and can be divided into IgG4-RD and non-IgG4-RD [1].

Constitutional inflammatory symptoms such as weight loss, fatigue, etc. can be seen but are not always present. Back pain is a common complaint; the pain is usually located at the lower back, abdomen, or flank. It is usually dull and transiently responds to nonsteroidal anti-inflammatory drugs (NSAIDs). The diagnosis can be made using CT or MRI, and contrast is often not needed; however, contrast can help to differentiate active and inactive lesions. There are currently no guidelines for indications of biopsy, but it’s often performed and can help rule out neoplastic RPF. High-dose glucocorticoids are the first-line treatment of retroperitoneal fibrosis. However, some conditions of IgG4-RD, such as IgG4-RD lymphadenopathy are subclinical and asymptomatic for years and only require watchful waiting. Rituximab can be used as second-line therapy in patients with glucocorticoid-resistant disease. It can also be used as the preferred first-line therapy in patients at high risk for glucocorticoid toxicity and patients with immediately organ-threatening disease [1]. Other drugs that can be used include, tamoxifen, mycophenolate, azathioprine, and medroxyprogesterone. C-Reactive Protein (CRP), erythrocyte sedimentation rate (ESR), and renal function should be assessed every four-eight weeks to monitor treatment response. Imaging can be repeated every three months and every six months interval once the disease has stabilized. Surgery can be considered in selected cases if medical management has failed [2].

## Case presentation

A 45-year-old male presented with complaints of insidious onset, worsening, pressure type, constant, lower back pain radiating to the flank for the past six months. Standing for a long period of time worsens the pain; the pain is not alleviated with over-the-counter analgesics or rest. As the patient’s back pain kept worsening, the patient noticed syncopal and presyncopal episodes every time he bends forward. Notably, six months prior to the onset of his current symptoms, he was also diagnosed with new-onset hypertension, for which he was started on losartan 25 mg daily.

The physical examination was notable for lumbar tenderness more prominent on the left side. There was no associated abdominal or costovertebral angle tenderness. 

**Figure 1:**
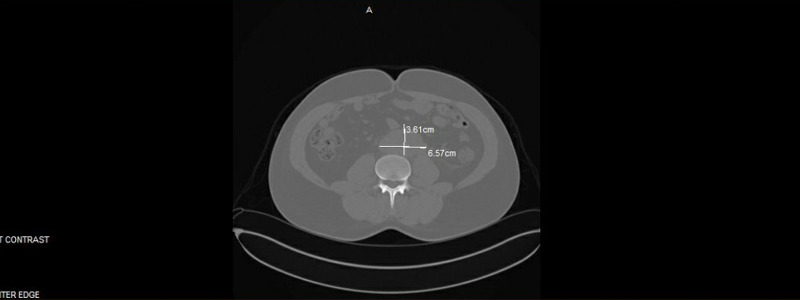
Retroperitoneal mass Large infiltrative retroperitoneal mass encasing the aorta and IVC measuring approximately 6.6 x 3.6 cm. It extends to the level of the aortic bifurcation. IVC: inferior vena cava

Routine bloodwork in the office as part of the annual well visit revealed a serum creatinine of 1.50 mg/dl (0.76-01.27 mg/dl), blood urea nitrogen (BUN) 20 mg/dl (6-24 mg/dl), with a glomerular filtration rate (GFR) of 55 ml/min/1.73 (>59 ml/min/1.73). Urinalysis revealed calcium oxalate crystals and 1+ proteinuria. A renal ultrasound was then ordered to further assess the kidney dysfunction. The ultrasound revealed multiple bilateral renal cysts, slight increased renal cortical echogenicity, suggesting medical renal disease, mildly enlarged prostate, and a hypoechoic mass surrounding the distal abdominal aorta but a normal renal Doppler evaluation. Subsequently, a non-contrast CT scan of the abdomen was done which revealed an infiltrative retroperitoneal mass encasing the aorta and the inferior vena cava (IVC) (Figure 1).

It also showed moderate left hydronephrosis and proximal hydroureter secondary to mass-effect on the proximal to the mid-left ureter and mesenteric lymphadenitis. Associated with it, left renal cortex thinning was found indicative of a long-standing obstruction.

The retroperitoneal mass has a wide variety of differentials, including malignancies. Tumor markers were then ordered. The patient was also referred to urology for evaluation of the hydronephrosis and hydroureter and the possibility of a stent placement. Although urology agreed that the patient needed a ureteral stent, a cystoscopy was first ordered to evaluate the patient’s anatomy. Interventional radiology (IR) was also consulted for the biopsy of the mass; it was discussed that the patient will need CT-guided biopsy of the retroperitoneal mass. However, an abdomen/pelvis MRI was ordered to clearly define structures around the mass. The abdomen/pelvis MRI revealed a small hemorrhagic cyst within each kidney and left periaortic mass. The tumor marker results were as follow: carcinoembryonic antigen (CEA) 0.9 ng/ml (0.0- 4.7 ) ; alpha-Fetoprotein (AFP) 1.5 ng/dl (0.0- 8.3 ) ; carbohydrate antigen CA19-19 27 U/ml ( 0- 35 ); cancer antigen CA125 5.4 U/ml ( reference range not established ); beta-human chorionic gonadotropin (beta-hCG) <1 mIU/ml (0-3 ). 

## Discussion

The patient’s clinical and radiologic pictures fit the diagnosis of retroperitoneal fibrosis. Our patient’s mass is periaortic, associated with hydronephrosis and mesenteric lymphadenopathy, without radiologic findings of intralesional fat or necrosis.

Retroperitoneal fibrosis can be idiopathic or secondary. Our patient did not have any risk factors for secondary RPF. The QuantiFERON gold test was negative, he has never smoked and does not recall working in an environment that would expose him to asbestos. The mass biopsy later on confirmed the diagnosis of IgG4 positive retroperitoneal fibrosis. The patient will receive high-dose steroids as well as stenting of the ureter.

Idiopathic RPF represents about 70 percent of all cases and can be IgG4 related or non-IgG4 related. Secondary RPF has been associated with various conditions, such as malignancies (carcinoid tumor, lymphomas, sarcomas, colorectal carcinoma, breast carcinoma, etc.), smoking, asbestos exposure, retroperitoneal hemorrhage, tuberculosis, histoplasmosis, radiation therapy, surgeries (lymphadenectomy, colectomy, aortic aneurysm repair), ergot alkaloids, dopamine agonists, biological agents such as etanercept or infliximab [3]. 

If it is missed, it may be life-threatening or organ damaging to the patient. Other symptoms can be related to anatomic involvement of the mass, for example, testicular swelling and pain can be seen with encasement of the spermatic vein by the RPF. Ureteral colic can be felt with the involvement of the ureter and subsequent hydroureter and hydronephrosis will develop; inferior vena cava compression can manifest as lower limb edema and possibly syncopal episodes [4]. Acute phase reactants (i.e., CRP, ESR) are usually elevated at the time of diagnosis and can be used to monitor disease activity but do not correlate with treatment response as relapse can occur in patients with normal values of the acute phase reactants [1-2]. Other lab values that have been reported in RPF include antinuclear antibodies (ANA) (up to 60 percent of cases) and alkaline phosphatase; plasma electrophoresis may show hypergammaglobulinemia. Normocytic normochromic anemia can also be found [2].

Numerous retroperitoneal cancers, infections, and non-neoplastic/noninfectious processes can mimic RPF. Non-neoplastic/noninfectious causes of low back pain can include lumbar disk disease, spinal stenosis, trauma, spondylolisthesis, osteoporosis, osteoarthritis, lumbar adhesive arachnoiditis, immune disorders such as ankylosing spondylitis, rheumatoid arthritis, irritable bowel disease, etc. [5]. Malignancies such as retroperitoneal leiomyosarcoma often involve the inferior vena cava and can present as a mass and/or lower extremity swelling on imaging; there is usually intralesional necrosis. Retroperitoneal liposarcomas can display intralesional fat, myxoid stroma (myxoid liposarcoma), or necrosis (pleomorphic liposarcoma) [6]. Retroperitoneal malignancies have a high propensity for metastases which are often present at the time of diagnosis or shortly after the diagnosis. Retroperitoneal lymphomas display a mantle growth pattern on imaging similar to RPF and can also present with associated hydronephrosis/ However, they can be differentiated from RPF on the basis of their size [6]. Infectious processes such as tuberculosis and actinomycosis should also be ruled out; the latter should especially be suspected in women with a history of intrauterine device use. Erdheim-Chester disease is another pathology that displays mantle growth on imaging, with bilateral involvement of tissues surrounding the aorta [6].

Typical workup includes acute phase reactants, IgG4 levels, renal function tests, imaging such as CT or MRI. Secondary causes of RPF need further evaluation. Serum protein electrophoresis is required in cases of suspected dysproteinemias [2]. 

## Conclusions

Back pain is a common complaint amongst young adults, with retroperitoneal fibrosis being a rare etiology. It should be suspected in patients with back pain and renal dysfunction, elevated creatinine, decreased urine output, or lumbar swelling on examination. It is important to identify this particular cause as it is treatable and may be easily missed. The workup includes kidney function assessment and abdominal imaging using CT or MRI. Biopsy of the mass helps identify malignancy. The treatment majorly comprises high-dose glucocorticoids. Renal function is continued to be monitored periodically. Imaging is performed every three-six months. If refractory, surgical options may be considered.

In summary, increasing awareness of this disease and increased interdisciplinary care are the key to the early diagnosis and treatment of RPF and to avoid complications.
